# Environmental Change Enhances Cognitive Abilities in Fish

**DOI:** 10.1371/journal.pbio.1000351

**Published:** 2010-04-06

**Authors:** Alexander Kotrschal, Barbara Taborsky

**Affiliations:** 1 Behavioral Ecology, Institute of Ecology and Evolution, University of Bern, Bern, Switzerland; 2 Evolution and Ecology Program, International Institute of Applied Systems Analysis, Laxenburg, Austria; Cambridge University, United Kingdom

## Abstract

Flexible or innovative behavior is advantageous, especially when animals are exposed to frequent and unpredictable environmental perturbations. Improved cognitive abilities can help animals to respond quickly and adequately to environmental dynamics, and therefore changing environments may select for higher cognitive abilities. Increased cognitive abilities can be attained, for instance, if environmental change during ontogeny triggers plastic adaptive responses improving the learning capacity of exposed individuals. We tested the learning abilities of fishes in response to experimental variation of environmental quality during ontogeny. Individuals of the cichlid fish *Simochromis pleurospilus* that experienced a change in food ration early in life outperformed fish kept on constant rations in a learning task later in life—irrespective of the direction of the implemented change and the mean rations received. This difference in learning abilities between individuals remained constant between juvenile and adult stages of the same fish tested 1 y apart. Neither environmental enrichment nor training through repeated neural stimulation can explain our findings, as the sensory environment was kept constant and resource availability was changed only once. Instead, our results indicate a pathway by which a single change in resource availability early in life permanently enhances the learning abilities of animals. Early perturbations of environmental quality may signal the developing individual that it lives in a changing world, requiring increased cognitive abilities to construct adequate behavioral responses.

## Abstract 2

Cichlid fish subjected to a single change in food ration early in life show enhanced learning abilities during juvenile and adult stages.

## Author Summary

Animals with higher cognitive abilities should be better capable of producing new, modified, or innovative behaviors as this ability could allow them to cope better with unpredictable environmental changes. Changing environments may hence select for higher cognitive abilities. Similarly, changing conditions during ontogeny can cause plastic responses, helping individuals to adapt to their current environment. In this study, we have used the cichlid fish *Simochromis pleurospilus* to show experimentally that individuals subjected to a change in food ration early in life (i.e., low to high or vice versa) outperform fish kept on constant rations in a learning task later in life. Remarkably, this result was independent of the direction of the implemented change or the average amount of food each fish received, and the results in the juvenile stage did not change in adulthood. Our results suggest that a single environmental change early in life might enhance cognitive abilities in animals.

## Introduction

The ability of adapting to changes in the environment is an important driving force of evolution, as recognized already by Darwin in his famous quote: “It is not the strongest of the species that survives…it is the one that is the most adaptable to change” [1]. Animals may adapt by altering their behavior, physiology, or morphology. The construction of behavioral responses is thought to be the fastest and most flexible way of adapting to new situations. Animals often have to deal with new situations for which they must devise novel or flexible solutions [2]. Field observations and laboratory studies showed that the advantages of novel or altered behaviors increase with the complexity of the environment (reviewed in [3],[4]). This suggests that frequent and unpredictable environmental changes may select for increased cognitive abilities allowing animals to meet these challenges by constructing adequate behavioral responses. In mockingbirds, for example, the complexity of songs is assumed to reflect their cognitive abilities, and species inhabiting areas with a low predictability of climatic patterns show more elaborate song displays than species in stable environments [5].

On the level of the individual, environmental instability can be encountered by plastic trajectories of the development of cognitive abilities. Environmental fluctuations early in life are known to enhance the behavioral flexibility of animals with regard to predator avoidance strategies [6],[7], feeding performance [7], and social behavior [6],[8]. A possible explanation for these behavioral effects is that variable environments evoke repeated neural stimulations resulting in faster and better learning [7]. Several studies showed that neural stimulation over longer periods by exposing animals to enriched environments (e.g., [9],[10]) can enhance brain development [5],[11], for example through an increased synaptic density [12], and can lead to improved learning abilities and memory capacity [12]. A food manipulation experiment indicated that a single change of diet can constrain neural development if later cognitive abilities are traded against the benefits of a compensatory growth response [7],[13]. On the contrary, an environmental change early in life should be expected to favor enhanced cognitive development, if this early perturbation signals the developing individual that it lives in a more variable environment. In response to this signal animals should develop increased cognitive abilities, which help them to construct adequate behavioral responses to the environmental challenges. An experimental evaluation of this hypothesis has been hitherto lacking.

Individuals of the African cichlid *Simochromis pleurospilus* live in a stable environment, but parts of the population experience a habitat shift around maturation [14]. If increased cognitive performance confers a fitness advantage when shifting habitats, we should expect that improved cognitive abilities can be triggered in *S. pleurospilus* by experimentally varying their juvenile environmental quality. We tested this prediction by investigating how the performance in a learning task was influenced by different juvenile feeding regimes in *S. pleurospilus*. Fish were fed either on a stable high or a stable low food ration, or rations were switched from low to high or vice versa. We trained the fish to associate a visual cue with food and tested how often they selected the positive stimulus. We tested their cognitive performance twice, at the end of the juvenile period and 1 y later when the fish were adults. We adhere to the broad definition of “cognition” as comprising “all mechanisms that invertebrates and vertebrates have for taking in information through the senses, retaining it, and using it to adjust behavior to local conditions” [15].

## Results

**Figure 1:**
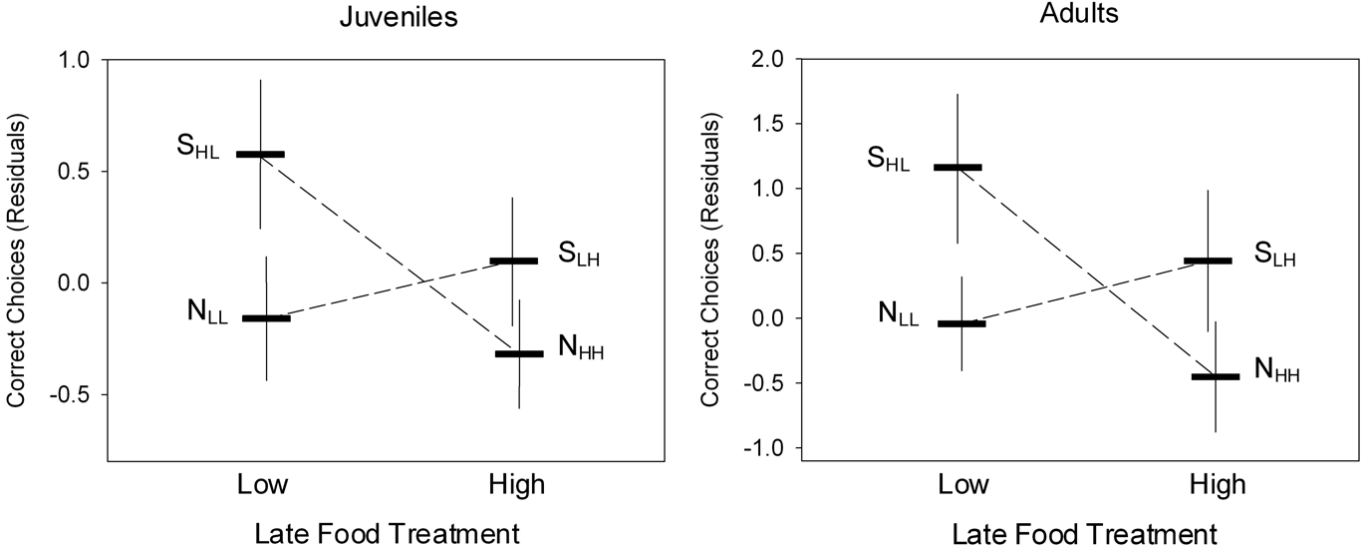
Fish on a switched diet have a superior learning performance. Relationship between early nutrition and associative learning performance in juvenile (left panel) and adult (right panel) *Simochromis pleurospilus*. In both tests neither early nor late resource availability influenced later learning performance. The interaction between treatments was significant, however, indicating that animals which experienced a switch between treatments during their upbringing outperformed those fed on constant rations. Left panel: mean residuals (± SE) of correct choices after accounting for the number of tests fish participated in; right panel: mean residuals (± SE) of correct choices after accounting for participation in tests and for previous testing experience (see Statistical Analyses for details). Experimental food treatments: N_HH_ (high food treatment), N_LL_ (low food treatment), S_HL_ (switched from high to low food), and S_LH_ (switched from low to high food).

The tests of learning abilities yielded similar results in juveniles (J) and adults (A). Neither the amount of food received before the switch (J: *p* = 0.62, A: *p* = 0.53) nor after the switch (J: *p* = 0.21, A: *p* = 0.12) influenced the number of correct choices significantly. The interaction between early and late food treatment was significant (J: *p* = 0.029, A: *p* = 0.005, Table 1) however, demonstrating that fish that had experienced a switch in feeding regime outperformed those fed constant rations. This effect is independent of the direction of the diet change (high-to-low or low-to-high; Figure 1).

**Table 1:** Cognitive performance of juvenile and adult *Simochromis pleurospilus* in dependence of food ration.

	Juveniles			Adults		
Variable	Wald χ^2^	*df*	*p*	Wald χ^2^	*df*	*p*
Intercept	63.4	1	<0.001	46.3	1	<0.001
Tested as juvenile	-	-	-	6.11	1	0.013
Early food treatment	0.24	1	0.620	0.29	1	0.529
Late food treatment	1.61	1	0.210	2.45	1	0.118
Early × Late Food Treatment	4.77	1	0.029	7.71	1	0.005

Using a binary probit-link GzLM, we tested the influence of early and late food treatment before and after the switches (fixed factors) on the total number of correct choices (dependent variable), including the number of trials the fish participated as the independent variable. Not all adults had previously been tested; therefore, whether or not the fish had been tested as juveniles was included in the adult model to control for experience differences.

Alternatively, learning ability might be affected by the average amount of food during the juvenile period. In that case we should expect the learning performance to increase linearly with food ration or, if an optimal food level exists, to follow a dome-shaped relationship. To test for this possibility, we determined the average amount of food each fish consumed relative to its own body mass. However, the number of correct choices was not related to mean relative food ration, neither with a linear (GLzM: J: *p* = 0.59, A: *p* = 0.86) nor with a quadratic predictor (GzLM: J: *p* = 0.26, A: *p* = 0.90).

**Figure 2:**
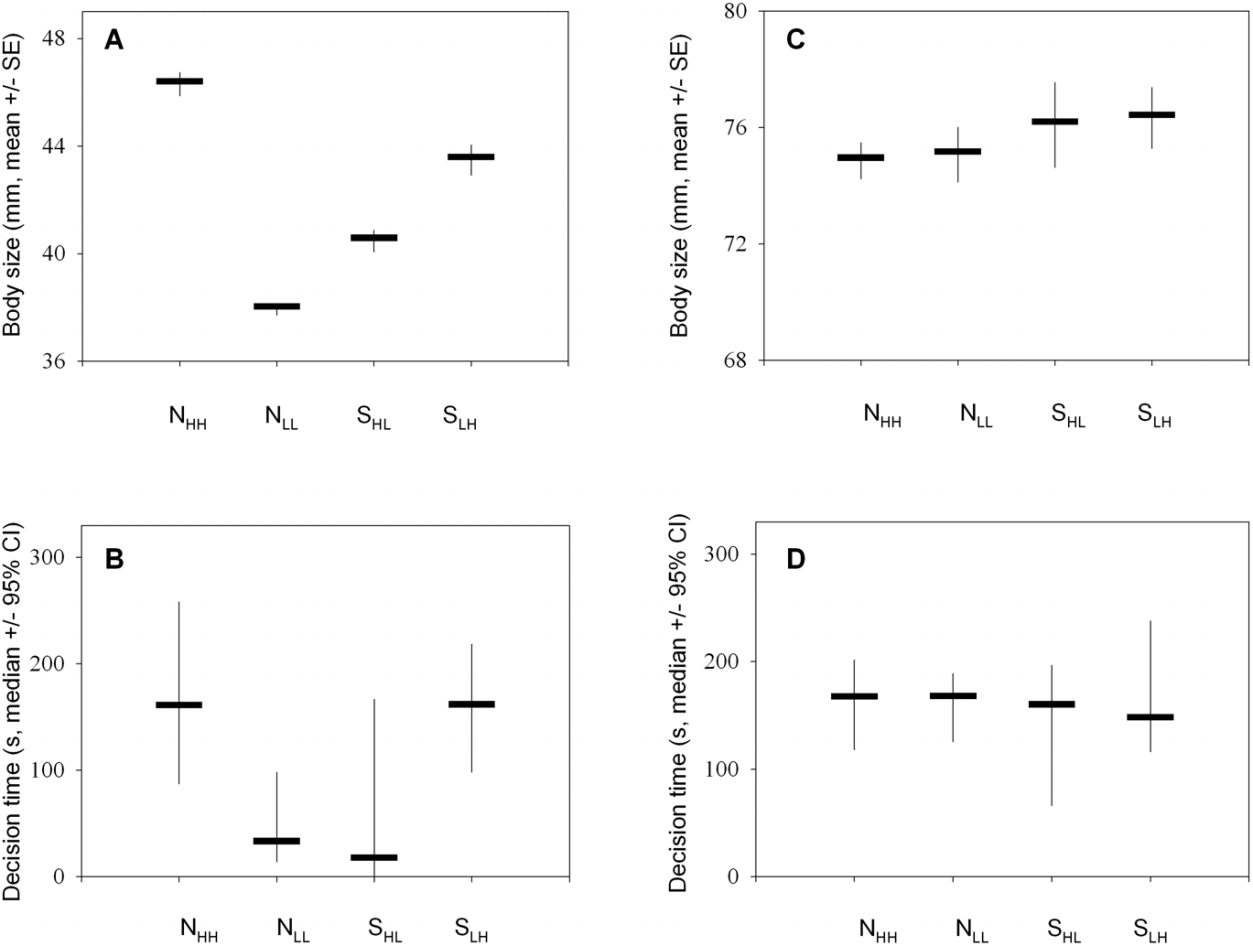
Food availability determines body size and latency times in juveniles. Mean body sizes during the experiment and median latency times from presentation of the stimulus until the fish left the shelter. During the cognition tests of juveniles the treatment groups differed in size (A). They were still subject to different feeding rations, which is likely to explain the substantial differences in decision latencies: N_HH_ and S_LH_ fish took significantly longer to enter the choice area than S_HL_ and N_LL_ fish (B). These differences had disappeared by the time adults were tested, since at this stage all fish were of similar size (C), received similar feeding rations eliminating potential motivational differences, and consequently had similar decision latencies (D). Experimental food treatments: N_HH_ (high food treatment), N_LL_ (low food treatment), S_HL_ (switched from high to low food), and S_LH_ (switched from low to high food).

During the test of juvenile cognitive abilities the animals of different treatment groups differed in body size (overall difference: ANOVA, *df* = 3, *F* = 103.88, *p*<0.001; differences between individual groups: Tukey's hsd test, all *p*<0.001, Figure 2A) and in the latency times to approach the stimulus (ANOVA, *df* = 3, *F* = 7.81, *p*<0.001; differences between individual groups: Tukey's hsd test, all *p*<0.01, Figure 2B). These differences had disappeared by the time the fish were tested for a second time during adulthood (size: ANOVA: *df* = 3, *F* = 0.61, *p* = 0.61, Figure 2C; latency time: ANOVA: *df* = 3, *F* = 0.38, *p* = 0.77, Figure 2D).

## Discussion

Individual *S. pleurospilus* that had experienced a change in food ration early in life outperformed those fish kept on constant rations in a learning task, suggesting that changes in environmental quality triggered a better cognitive performance in these fish. Remarkably, this result was independent of the direction of the implemented change and the mean rations received. The difference in learning abilities between treatment groups remained constant between juvenile and adult stages of fish tested 1 y apart, which suggests that a single change in food availability can trigger better cognitive abilities probably for lifetime. Several alternative possibilities of how ration, size, or growth rates can affect cognition can be ruled out as likely explanations for our results.

### Current Food Ration

Juveniles differed in size across the treatment groups as they were subject to different feeding rations during the tests. N_HH_ and S_LH_ fish received near *ad libitum* food and were presumably satiated, whereas N_LL_ and S_HL_ fish experienced a food shortage most likely resulting in a stronger motivation of these two groups to approach the test apparatus. This is reflected in substantial differences in the times to leave the shelter and to approach the choice apparatus. A differential motivation cannot explain our results however, as in this case the learning performance should differ between N_HH_/S_LH_ and N_LL_/S_HL_ fish. Moreover, these differences in latency time had disappeared, when testing the fish the second time during adulthood. Potential motivational differences were now eliminated as the size differences had vanished and all fish received the same food rations.

### Poor Early Nutrition

Poor early nutrition can adversely affect neural development [16]–[18] and it can have a negative impact on song learning in birds [19],[20] and intelligence quotients in humans [21]–[23]. Also this factor cannot explain our findings as N_HH_ fish did not perform better than N_LL_ fish. We assume that the low-food ration was sufficiently high to sustain normal neural development, as in a previous study [14],[24] *S. pleurospilus* raised on the N_LL_ ration developed and reproduced normally.

### Growth Trajectories

Body size is amongst the traits under strongest selection [25]. Juvenile fish should have high incentives to grow fast, since the number of potential gape-size limited predators decreases exponentially with increasing body size [26]. Fast growth can have negative effects, however (reviewed in [27]), including a negative influence on cognitive performance. In zebra finches, birds that had the highest rates of compensatory growth after experiencing a period of a reduced food ration performed worst in a subsequent learning task [13]. This effect might result from a trade-off between investment in growth versus neural development [28],[29] or from prolonged stress due to increased foraging activity leading to chronically elevated levels of corticosterone, which in turn can adversely affect neural development [30]. If compensatory growth had affected the learning performance of *S. pleurospilus*, N_HH_ fish should have outperformed those groups that had not started on a high-food diet and that exhibited compensatory growth in our experiment (all fish reached similar sizes at the time when adults were tested, but N_LL_ fish took longer to do so than S_LH_ fish). If the brain was especially vulnerable to negative effects of compensatory growth during the juvenile period, then the fish that experienced a switch from a low to a high ration (S_LH_; highest degree of compensatory growth) should perform worst. The opposite was the case, however, as S_LH_ fish outperformed N_LL_ fish.

### Sequence Effect

To ensure that the learning experience of juveniles did not influence the subsequent learning performance of adults [31], we performed a test series, which confirmed that the fish did not remember the conditioned cue of the juvenile test series before starting the second series. Other fish species have been shown to forget learned foraging techniques already within 2 d [32], whereas the tests of juveniles and adults in our experiment were more than 1 y apart. We are therefore confident that we tested independent learning abilities of the fish in both test series rather than memory effects. Twelve individuals, which replaced fish that had died until the onset of the second test series and which had not been tested as juveniles, slightly outperformed the previously tested fish in the learning tests (Table 1; see Material and Methods for details). Possibly fish tested 1 y before may still have associated the test apparatus with a food reward, as apparently they were less afraid of the test apparatus (shorter latency times until approach; see Material and Methods). They may therefore have been less attentive to the type of stimulus cue during the training phase than previously untested fish. These fish approached the apparatus more cautiously and hence may have had more time to associate the cue with food. The effect of previous learning experience was the same across all treatment groups.

### Training

Environmental conditions during development may trigger changes in morphology, physiology, or behavior, which can confer an adaptive advantage later in life if an animal remains in these conditions [33]. The main mechanisms proposed to explain such plastic responses to environmental cues involve repeated stimulation, for example, through physical exercise facilitating muscle development or by early neural stimulation through environmentally enriched raising conditions, which enhances cognitive abilities later in life [7],[12],[34]. But neither environmental enrichment nor training through repeated neural stimulation can explain our findings, as the sensory environment was kept constant during ontogeny and resource availability was changed only once. Our results rather show that already a *single* event—a change of food ration—during early ontogeny triggers learning ability possibly indicating the existence of a novel pathway of plastic neural development.

It has been hypothesized that changing environments improve learning abilities, which consequently may allow animals to behave more adequately and flexibly [7]. Our results support this hypothesis by showing that environmental change can indeed directly affect learning abilities, independently of motivational differences between individuals. Changing environments experienced early in ontogeny can greatly improve the flexibility of behavior [6]–[8]. If such effects result partially from better learning abilities induced by early environmental change, these studies elucidate the manifold possible consequences of improved learning abilities, which extend to a wide range of behavioral contexts.

The life history and ecology of *S. pleurospilus* suggests that the improvement of cognitive abilities in response to environmental change is adaptive. *S. pleurospilus* are algae grazers and hence depend upon the primary production of turf algae, which is influenced mainly by light intensity and a suitable substrate such as rocks and stones [35]. Algae productivity decreases exponentially with depth [35]. While juvenile *S. pleurospilus* inhabit the shallow regions of the lake with the highest algae intensity and some fish stay there throughout adulthood, other fish start to settle in deeper water around maturation [14]. These fish should benefit from increased cognitive abilities, as they have to cope with entirely different nutritional conditions. Improved cognition may help them to find and remember patches of high-quality turf algae (reviewed in [36]), while those fish remaining stationary in the natal habitat do not necessarily require a better cognitive performance. Hence our findings suggest that habitat shifts can make these animals smarter. More generally, animals forced to cope with environmental changes as caused, for example, by anthropogenic perturbations of their habitats may benefit from improved cognitive abilities induced by these perturbations when forced to adjust to the new conditions.

In conclusion we show for the first time that a single change in food availability early in life can enhance life-long learning abilities. Hence our study provides experimental support for the hypothesis that selection favors higher cognitive abilities in unpredictable or changing environments [5],[9]. It also suggests a mechanism of how animals can acquire better abilities to cope with such environments: an environmental switch early in ontogeny may enhance learning ability persistently.

## Materials and Methods

### Study Species

*Simochromis pleurospilus* is a maternally mouthbrooding cichlid of the subfamily Tropheini endemic to Lake Tanganyika, East Africa. It lives along the rocky shores of the lake where it feeds on epilithic turf algae. *S. pleurospilus* reproduces all year round and adult males defend small, adjoining territories of 2–4 m^2^ where females visit them to spawn. Juveniles and females are non-territorial and use large home ranges. After spawning females leave the male territory immediately and care for the clutch on their own [24]. Approximately 28 d after spawning, the young are independent. Juveniles and adults live sympatrically, but juveniles are confined to the shallow areas between 0.5 and 2 m depth, whereas adults often disperse to feed in greater depth between 1 and 12 m ([14], A. Kotrschal & B. Taborsky submitted).

### General Experimental Methods

We raised 130 fishes in separate 20-l Plexiglas tanks, each equipped with a layer of sand, a flower-pot half for shelter, and an internal biological filter (see [24] for details on experimental set-up). The experimental fish were derived from seven clutches of different females, and siblings were proportionally distributed over all treatments. We exposed the fish to two different feeding conditions in early and late adolescence, respectively, using a full-factorial design. Fish either received (1) a high food ration both in early and late life (abbreviated as N_HH_, where “N” stands for “Not switched”; *n* = 40); (2) a low food ration both in early and late life (N_LL_, *n* = 40); (3) a high food ration in early life, switched to a low food ration in late life (S_HL_, where “S” stands for “Switched”; *n* = 22); or (4) a low food ration in early life, switched to a high food ration in late life (S_LH_, *n* = 22). Diet switches were performed either at 77 d (i.e., after the first third of the juvenile period; S_LH_: n_77d_ = 11; S_HL_: n_77d_ = 11) or at 133 d of age (i.e., after the second third of the juvenile period; S_LH_: n_133d_ = 11; S_HL_; n_133d_ = 11). We had switched diets at two different ontogenetic stages to enhance the chances to capture a potential sensitive period when a change in resource availability affects cognitive abilities. As in the learning trials fish switched at day 77 did not perform differently from fish switched at day 133, neither as juveniles (GzLM; S_LH_: 77 d versus 133 d: χ^2^ = 2.1, *p* = 0.15; S_HL_: 77 d versus 133 d: χ^2^ = 0.3, *p* = 0.57) nor as adults [S_LH_: 77 d versus 133 d: χ^2^ = 0.09, *p* = 0.77; S_HL_: 77 d versus 133 d: χ^2^ = 2.3, *p* = 0.13 (details of model see section Statistical Analysis)], we combined the data of early and late switched fish resulting in four treatment groups: N_HH_, N_LL_, S_LH_, and S_HL_. Fish were fed 6 d a week with standardized agarose cubes containing an amount of Tetramin flake food corresponding to 12% (near ad lib) or 4% of mean body weight plus 5% *Spirulina* algae. All fish of a treatment group received the same food ration, which was based on the mean body mass of fish within this group. We adjusted the food rations to increasing mean body weights every 14 d. We stopped adjusting the rations to body weight in N_HH_ fish at 189 d, because they no longer depleted the food cubes. We continued to adjust the ration for the N_LL_, S_LH_, and S_HL_ fish until day 259 when they reached the same body size as N_HH_ fish. Thereafter all fish were kept on the same food ration. We measured lengths and weights of fish every 3 wk. Standard lengths were read from a measuring board with a 1 mm grid and were estimated to the nearest 0.5 mm by eye. Weights were read to the nearest 1.0 mg from an electronic balance. All measurements were taken before the daily feeding and done by the same person (A.K.).

### Learning Tests

We first trained the animals to associate a certain visual cue with food and thereafter determined the number of correct decisions made when presenting the cue. We did the first test series in the juvenile phase shortly before maturation (J) at an age of 172 d (±10 d) when the fish still received different food rations and differed in body size between treatments. The second test series was done 1 y later in adults (A) at an age of 585 d (±10 d), when all fish were fed the same rations and were of similar size. Each fish was tested in its individual raising tank.

#### Experimental set-up

**Figure 3:**
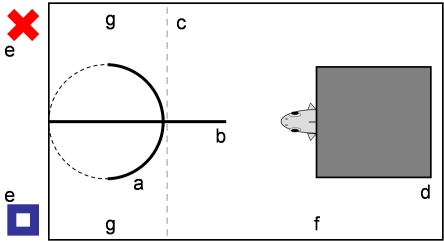
Test of cognitive performance. Schematic representation of the set-up used to test the cognitive abilities of *Simochromis pleurospilus*. Letters refer to “a” choice tube with cut out (dotted line), “b” PVC plate, “c” clear rise able sheet of Plexiglas, “d” elevated filter used as shelter, “e” stimulus cues, “f” neutral area, and “g” choice area.

All tanks were equipped with a test apparatus (Figure 3) consisting of a 25 cm, grey PVC tube with 8 cm diameter (“a”) that was divided in half by a PVC plate (“b”, Figure 3). We created an entrance to the tube by removing a half-circular, 3 cm high piece of PVC at the lower end of the tube. The tube was placed upright against the front pane of the respective tanks and oriented such that the entrance (dotted line, Figure 3) faced the front pane of the tank. To prevent the fish from using the experimental tube as shelter, we blocked the access with a clear sheet of Plexiglas (“c”). We removed the flower-pot shelter and instead placed the biological filter on two 5 cm high granite cubes to produce a central crevice for the fish (“d”).

#### Training phase

During the training phase, two visual cues, a red cross and a blue square, were presented simultaneously on either side of the tube outside of the tank (“e”) such that the fish hiding underneath the filter could see both cues. The experimenter dropped the food pellet visibly in front of the rewarded stimulus (pellets are negatively buoyant). Then the Plexiglas divider was remotely lifted 5 cm off the ground and the fish was able to enter the choice area and to access the food. Half of each treatment group was haphazardly chosen to be conditioned to the red cross and the other half to the blue square. Training trials were repeated twice a day for 7 d. The side where the positive stimulus was placed during each training session was decided by throwing a dice.

#### Test phase

During the test trials we followed a similar procedure as in the training phase, only now the experimenter dropped the food pellet *inside* the tube to be invisible to the fish. To rule out that the fish follow olfactory cues, we tested whether they leave the shelter and feed on the pellet when it was dropped inside the tube without concurrent presentation of the stimulus. In 12 tests, however, none of the fish entered the choice area within 12 min. During the tests the experimenter was hidden behind a wall and observed the fish through a peephole. We recorded the latency times until the fish left the neutral shelter area (“f”) to enter the choice area (“g”) and the visual cues chosen by the fish. When the fish did not enter the choice area within 12 min, we blocked the entrance, removed the food pellet and the visual cues, and retested this fish 2–3 h later the same day. If the fish still did not enter the choice area within 12 min during the repeated trial, this was treated as missing value. After the test the food pellet was left in the tank for the fish to eat. Juveniles were tested once a day on 6 consecutive d by the experimenter Marc Steinegger. One year later, adults were tested once a day on 10 consecutive d by the experimenter A.K. following the same training procedure and testing protocol. Due to time constraints (13 h of light per day), we were able to test a maximum of 90 fish within 1 d. For the first test series we trained and tested all juveniles that had experienced a food switch (22 S_LH_, 22 S_HL_) and a sub-sample of N_HH_ (23) and N_LL_ (23) fish.

#### Repeated test series

To determine whether the fish had remembered the learning task from the first (J) until the second test series (A), we tested their performance three times without food before starting with the second training trials. None of the treatment groups performed above chance level. For the test series with adult fish, we haphazardly assigned the rewarded visual cues such that in all treatment groups half of the fish were conditioned to the same cue as in the juvenile test series and half were conditioned to the other cue.

### Sample Size

Twelve juveniles were excluded, as they were used in a pilot study after which we adjusted the testing protocol. Furthermore 12 fish never left their shelter within 12 min, yielding a final sample of 66 fish for the juvenile test series (N_HH_ = 19, N_LL_ = 19, S_HL_ = 14, S_LH_ = 14). One year later some fish that had been tested previously had died in the meantime. Therefore we added 12 previously untested individuals to increase sample sizes. We used all S_HL_, S_LH_, and N_LL_ fish still alive and 30 N_HH_ fish for the second test run. Five adults refused to take food from the test apparatus and eight adults never left their shelter within 12 min, resulting in a sample size of 77 fish for the adult test series (N_HH_ = 30, N_LL_ = 25, S_HL_ = 8, S_LH_ = 14). Overall 46 fish participated in all 6 juvenile trials, and 69 fish participated in all 10 adult trials. The mean rate of participation was 5.3 (±1.4 SE) times out of 6 in juveniles and 9.7 (±1.0 SE) times out of 10 in adults. Although juvenile N_LL_ and S_HL_ fish participated more often than N_HH_ and S_LH_ fish (ANOVA: *F* = 4.63, *p* = 0.005), this did not bias the results because the statistical model accounts for participation rate (see Statistical Analysis). Adult fish of all treatment groups participated at a similar level (ANOVA: *F* = 1.04, *p* = 0.38).

### Statistical Analyses

Since not all fish participated in every trial we used binary probit-link generalized linear models (GzLM) to analyze the cognitive performance with the total number of correct choices as the dependent variable and the number of times the fish participated as the independent variable [37]. We included food ration in early adolescence (“early food treatment”) and in late adolescence (“late food treatment”) as fixed factors. Twelve adults that had not been tested as juveniles took on average 70 s longer to enter the choice area (Mann-Whitney U: Z = −2.19, *p* = 0.028), but they outperformed those fish already tested as juveniles (GzLM, χ^2^ = 6.11, *p* = 0.013). As the latter effect occurred across treatment groups (indicated by an absence of a significant interaction between treatment group and previous test experience, GzLM: χ^2^ = 352, *p* = 0.84), we included previous test experience (yes or no) in the model of adult learning performance.

To examine whether the amount of food per se influenced the likelihood of correct choices, we tested if a positive, a negative (linear predictor), or a dome-shaped (quadratic predictor) relationship exists between these two variables. We determined the amount of food received by each individual by calculating the percentage of food mass contained in the food pellets relative to the body mass of individuals using data from our tri-weekly body mass measurements. We then took the mean of these values during the entire juvenile period (i.e., until week 30) as a measure of food consumed by individual fish. All analyses were done with SPSS 17.0, SPSS Inc., Chicago.
